# Supplementary material for: Sorting at embryonic boundaries requires high heterotypic interfacial tension
Source: Nat Commun. 2017 Jul 31;8:157. doi: 10.1038/s41467-017-00146-x (PMC5537356; doi:10.1038/s41467-017-00146-x)
Supplement: Supplementary file 2 — Supplementary Software 1 [file 41467_2017_146_MOESM2_ESM.zip › PottsModel/SrcPottsModel/doc/model/class-use/Constants.Manager.html]

Uses of Class model.Constants.Manager


---


|  |  |  |
| --- | --- | --- |
| **Overview** \| **Package** \| **Class** \| **Use** \| **Tree** \| **Deprecated** \| **Index** \| **Help** | |  |
| PREV   NEXT | **FRAMES**    **NO FRAMES**     **All Classes** |  |


---


## **Uses of Class model.Constants.Manager**

| Packages that use Constants.Manager | |
| --- | --- |
| **model** |  |

Uses of Constants.Manager in model

| Subclasses of Constants.Manager in model | |
| --- | --- |
| `private  class` | `Constants.GuiManager`             Manages graphical output information related to this simulation. |
| `private  class` | `Constants.LatticeManager` |
| `private  class` | `Constants.MetaManager`             Manages meta and statistical information related to this simulation. |
| `class` | `Constants.ParameterManager`             Manages energy information related to this simulation. |
| `private  class` | `Constants.PathManager`             Manages file paths. |

---


|  |  |  |
| --- | --- | --- |
| **Overview** \| **Package** \| **Class** \| **Use** \| **Tree** \| **Deprecated** \| **Index** \| **Help** | |  |
| PREV   NEXT | **FRAMES**    **NO FRAMES**     **All Classes** |  |


---
